# Impact of Socioeconomic Factors on Patient’s Adherence to Lifestyle Modifications Aimed at Reducing Future Fracture Risk in Geriatric Patients With Peritrochanteric Fragility Fractures of the Hip

**DOI:** 10.7759/cureus.23807

**Published:** 2022-04-04

**Authors:** Jaiman Sharma, Sachin Sonawane, Janapamala V S Kishore, Rajesh Pareek, Mukund Pai

**Affiliations:** 1 Orthopaedics and Traumatology, Dr D.Y Patil Medical College and Hospital, Pune, IND; 2 Orthopaedics and Trauma, Dr D.Y Patil Medical College and Hospital, Pune, IND; 3 Orthopaedics, Dr D.Y Patil Medical College and Hospital, Pune, IND; 4 Orthopaedic Surgery, Dr D.Y Patil Medical College and Hospital, Pune, IND

**Keywords:** public health policy, patient adherence, fragility fractures, osteoporotic fractures, secondary fracture prevention

## Abstract

Introduction

A history of fracture is a well-documented risk factor for sustaining future falls and subsequent fractures in geriatric patients. Orthopedic surgeons advocate various lifestyle modifications to reduce the risk of sustaining a recurrent fracture in this vulnerable group. However, it has been observed that patients seldom adhere to this advice and the rate of fragility fractures has thus continued to rise in this vulnerable subset of the population. The factors influencing the compliance of patients with various modifications have not been documented in any previous studies. In our study, we aimed to evaluate the factors influencing patient adherence to various lifestyle modifications advised by orthopedic surgeons for reducing future fracture risk.

Material and methods

A total of 112 patients aged >65 years who were diagnosed as having a peritrochanteric fragility fracture of the hip and were treated operatively for the same were included in this study. Upon discharge from the hospital, the patients were advised 10 lifestyle modifications to reduce the recurrent fracture risk. A data collecting form that graded the adherence on a 20-point scale (2 points for each lifestyle modification) was prepared by the investigators. Upon the six-month follow-up visit, adherence was assessed on the 20-point scale, and data were collected via the face-to-face interview method. Statistical analysis was accomplished by the Chi-square test and logistic regression analysis.

Observations and results

Of the 112 subjects included in the study, 58 (51.7%) were male and the mean age was 75 ± 8 (65 - 92) years. The adherence to less than 4 recommendations (Score <8) was seen in 39.2%, adherence to 4 - 6 recommendations (Score between 8 - 12) was seen in 30.86%, adherence to 6 - 8 recommendations (Score between 12 and 16) was seen in 29.94% and adherence to eight to 10 recommendations (score between 16 and 20) was seen in 0% of participants. According to the regression analysis, the presence of adherence to less than six recommendations was related to the low-income level (OR=0.298; 95%CI - 0.132-0.666; p<0.001) and lack of education and awareness (OR=2.329; 95% CI - 1.114-4.859; p=0.002).

Conclusion

The rates of adherence to advised lifestyle modifications were generally found to be low. Compliance was particularly reduced in patients belonging to the lower socioeconomic strata, which were less likely to be educated and had lower rates of income. The authors concluded that merely advising lifestyle modifications was not enough, and various social and public health measures are required to improve patient compliance, with the broader aim of ending the menace of recurrent fragility fractures.

## Introduction

Fractures of the hip in the elderly have been recognized in literature since time immemorial. Reduced bone mineral density, reduced propensity for protective muscle reflexes, and conditions such as cataracts, parkinsonism, Alzheimer’s, and many more age-related ailments further compound the problem [1]. Centre JR et al. studied the risk of sustaining a refracture after sustaining a low trauma fracture and reported that the risk of sustaining a fall and subsequently a fracture increases by two-fold in women and nearly four-fold in men, and this increased risk persisted for at least 10 years [2]. This finding was supported by multiple studies that ascertained that a history of hip fractures increased the future fracture risk.

Recognizing the menace of fragility fractures in the elderly, surgeons and public healthcare providers tried to come up with strategies in an attempt to reduce the risk of falls and fractures in this vulnerable cohort of patients [3]. Various modalities were postulated, which included prescription of regular calcium and vitamin D supplements, regular exercise and physiotherapy programs, and the use of a walking cane/stick amongst others. These modalities work by increasing the bone mineral density, improving the balance of the body, and reducing slippage. While these modalities are known to work, no study has reliably studied the compliance of patients to these commonly prescribed and advised modalities aimed at reducing the risk of falls and fractures. Lack of uniformity amongst healthcare provider prescriptions, lack of available data on compliance, and poor follow-up are to blame for the absence of reliable data in this regard.

In this study, we have attempted to study the compliance of patients to various lifestyle modifications, which are aimed at reducing future fracture risk. Furthermore, we have attempted to relate patient compliance with their socioeconomic status in an attempt to ascertain if the two are interrelated.

The findings of this study were presented to a panel as part of The 9th Fragility Fracture Network Congress and an abstract was published in the Journal of Geriatric Orthopedic Surgery & Rehabilitation in December 2021.

## Materials and methods

A cohort of 140 patients aged >65 years with a history of a fragility fracture of the hip (intertrochanteric or subtrochanteric fracture) was initially enrolled for the study. Out of this original cohort, 25 patients were lost to follow-up while three patients died during the course of the study as a result of their co-morbidities (hypertension leading to stroke in one and coronavirus disease 2019 (COVID-19) infection in two). Finally, 112 consenting adults were included in the study. Patients with pathological fractures were excluded from the study. Revision arthroplasty cases and those not consenting to the procedure were also excluded from the study.

Upon discharge, the patients were given a list of lifestyle modifications and were asked to incorporate 10 individual parameters with an aim of reducing falls and fracture risk.

The patients were followed up for a period of one year after being operated on for hip fractures in the outpatient department of Dr. D.Y Patil Medical College, Pune. The patients were asked to follow up every two months and on each visit, they were asked about their compliance with the lifestyle modifications. Their compliance was graded on a three-point scale (Table 1).

**Table 1:** Grading patient compliance on a three-point scale

S.No	Recommended Modification	Complete Adherence	Incomplete Adherence	No Adherence
1	Stopping alcohol and smoking	2	1	0
2	Balanced diet and weight optimization (BMI 18.5 – 24.9)	2	1	0
3	Regular weight-bearing exercises	2	1	0
4	Wearing protective hip pads	2	1	0
5	Use of a walking stick/cane.	2	1	0
6	Installing grab bars in the bathroom	2	1	0
7	Installing anti-skid flooring in the house	2	1	0
8	Taking regular calcium and Vitamin D supplementation	2	1	0
9	Taking a six-month course of Inj. Teriparatide	2	1	0
10	Hiring domestic help for assistance	2	1	0
Total		20	10	0

The data were obtained by the face-to-face interview method. The education level of the patients was categorized into six levels: illiterate; literate but no graduation from any school; graduated from elementary school; graduated from junior high school; graduated from university; postgraduates. The income level of these patients was also graded as: Low (income is not sufficient for usual expenses); Intermediate (income level is equal to the usual expenses); High (income level exceeding the usual expenses).

Furthermore, the patients were asked if they lived alone, lived with their children, or lived with domestic help. Additionally, the patients were also categorized based on where they lived: Living in rural areas (tier 3 cities), living in semi-urban areas (tier 2 cities), and living in urban areas (tier 1 cities).

The data collected were analyzed using SPSS 22 software (IBM Corp., Armonk, NY) in a Microsoft Excel spreadsheet (Microsoft Corporation, Redmond, WA). The chi-square test was used to test the significance of quantitative data. After applying statistical principles, a p-value of 0.05 was determined to be statistically significant.

## Results

Of the 112 subjects included in the study, 58 (51.7%) were male and 54 were female (48.21%). The mean age was 75 ± 8 (65-92) years. The adherence to less than four recommendations (Score <8) was seen in 39.2%, adherence to four to six recommendations (score between 8 and 12) was seen in 30.86%, adherence to six to eight recommendations (score between 12-16) was seen in 29.94% and adherence to eight to 10 recommendations (Score between 16 and 20) was seen in 0% of participants. According to the regression analysis presence of adherence to less than six recommendations was related to the low income level (OR=0.298; 95%CI - 0.132-0.666; p<0.001) and lack of education and awareness (OR=2.329; 95% CI - 1.114-4.859; p=0.002). The rate of compliance to individual parameters was subsequently charted (Table 2).

**Table 2:** Rate of compliance to original parameters

S.No	Recommended Modification	Complete Adherence	Incomplete Adherence	No Adherence
1	Stopping alcohol and smoking	30 (26.7%)	69 (61.6%)	13 (11.6%)
2	Balanced diet and weight optimization (BMI 18.5-24.9)	42 (37.5%)	46 (41%)	24 (21.4%)
3	Regular weight-bearing exercises	43 (38.3%)	52 (46.4%)	17 (15.1%)
4	Wearing protective hip pads	19 (16.9%)	36 (32.1%)	57 (50.8%)
5	Use of a walking stick/cane	33 (29.4%)	67 (59.8%)	12 (10.7%)
6	Installing grab bars in the bathroom	30 (26.7%)	N/A	82 (73.2%)
7	Installing anti-skid flooring in the house	21 (18.7%)	N/A	91 (81.2%)
8	Taking regular calcium and Vitamin D supplementation	80 (71.4%)	17 (15%)	15 (13.3%)
9	Taking a six-month course of Inj. Teriparatide	20 (17.8%)	33 (29.4%)	59 (52.6%)
10	Hiring domestic help for assistance	18 (16%)	25 (22.3%)	69 (61.6%)
	Average compliance	29.94%	30.86%	39.20%

## Discussion

The incidence of fragility fractures is on the rise owing to a rise in the incidence of osteoporosis [4]. Pasco et al., in their landmark paper titled “human cost of fracture,” highlighted the financial burden that an unexpected fall and subsequent fracture can place on an individual [5]. Rising costs of healthcare add to this problem and when accounted for opportunity costs, fractures account for a substantial annual burden on individuals and healthcare institutions alike.

The risk of a future fracture risk increases by two to threefold in patients with a history of fractures as compared to the general population [2]. Considering these patients are already battling the physical and financial burden from the original injury, this repeated incidence leaves them belittled physically, mentally, and financially. This mused healthcare providers to come up with prevention strategies to prevent the risk of falls in this vulnerable population, and today, almost every orthopedic clinic advocates for one or the other modality to improve bone health and reduce the risk of falls with an ultimate aim of reducing the incidence of fragility fractures. It has been noted, however, that the incidence of fragility fractures has continued to rise in spite of surgeons and healthcare providers advocating for preventive measures. In 2007, Serour studied the impact of cultural factors on lifestyle measures and concluded that cultural factors influence compliance in both a positive and a negative manner [6]. Building on this study, the inverse relationship between rising advocates of fracture preventive measures and the rising incidence of fragility fractures can be explained by the fact that the patients do not adhere to the advised preventive measures and ultimately succumb to a fall and end up sustaining a repeat fracture. The reasons for this disparity are multifold and can be explained by various independent variables, which have an impact on patient adherence. These include the level of education, income level, and living conditions of the individual. Through this study, we were able to ascertain that patients who were educated fell into the intermediate and high-income categories, lived in urban areas, and had a higher level of adherence to preventive measures provided by their doctors. In stark contrast to this, in spite of regular home check-ups and reminders by social healthcare workers, compliance remained poor in patients who were uneducated, poor, and lived in urban areas.

Furthermore, a small category of educated patients living in urban areas still suffered from poor adherence because of loss of job, primarily because they were the primary bread earners of the family.

Another incidental finding from this study was that uneducated patients who lived in rural areas, belonged to the lower socioeconomic strata, and were taken care of by their families fared better with regard to adherence and future fracture risk when compared to educated patients who lived in urban areas and belonged to higher socioeconomic strata but were taken care of by their domestic help. This avenue offers exciting opportunities to research and study the approaches of care used by these care providers to better understand adherence to recommendations.

The rates of adherence to advised lifestyle modifications were generally found to be low. Compliance was particularly reduced in patients belonging to the lower socioeconomic strata, which were less likely to be educated and had lower rates of income. The authors concluded that merely advising lifestyle modifications to reduce fracture risk was not enough to stop the menace of recurrent fragility fractures in this vulnerable population group.

This study suffers from two important limitations. First, there are crucial literature gaps while addressing the compliance of patients to fracture prevention measures. While research has been focused on the prevention of various non-communicable diseases, little has been documented on the role of fracture prevention measures and patient compliance to them. Second, our study solely focuses on a cohort of patients based in a developing country like India. These findings can be corroborated in other developing countries, however, owing to the socioeconomic differences, the findings can be in stark contrast if such a study is carried out in underdeveloped parts of Africa or the developed nations in Europe. The authors feel that further research is the need of the hour to come up with a universally replicable and applicable fracture prevention program to address the menace of fragility fractures.

## Conclusions

There is an urgent need to institute social security and public health measures to improve patient adherence and reduce future fracture risk in an aging population. Furthermore, the role of caregivers is highlighted by the fact that patients living with their families fared better with regard to adherence to modifications as compared to patients living alone or with domestic help. Further qualitative studies into this can help in a better understanding of caregiver practices that improve patient compliance. From this study, orthopedic practitioners and public health workers can take away two crucial practice points. First, extending the incentives provided by government schemes and insurance packages to include provisions for certain preventive measures, such as a six-month supply of calcium supplements as part of the insurance scheme from registered pharmacies, will incur marginally high costs but will overall reduce the financial burden on the healthcare ecosystem as a whole. Second, installing anti-skid flooring and hand railings in public spaces, such as shopping malls and movie theaters, will reduce the risk of falls in these places and will be beneficial to society at large without an additional financial burden on the individual patient.
